# *Wolbachia*-centered cytoplasmic axis links elevational mitochondrial DNA turnover with microbiome restructuring

**DOI:** 10.1093/ismejo/wrag188

**Published:** 2026-07-17

**Authors:** Yong Zhang, Xueyu Huang, Qiangkun Li, Yuhang Ruan, Yanhua Long, Shouke Zhang, Yunqiu Yang

**Affiliations:** State Key Laboratory for Tea Plant Germplasm Innovation and Resource Utilization, Key Laboratory of Tea Biology and Tea Processing, Ministry of Agriculture and Rural Affairs, Anhui Agricultural University, Hefei, Anhui 230036, China; State Key Laboratory for Tea Plant Germplasm Innovation and Resource Utilization, Key Laboratory of Tea Biology and Tea Processing, Ministry of Agriculture and Rural Affairs, Anhui Agricultural University, Hefei, Anhui 230036, China; State Key Laboratory for Tea Plant Germplasm Innovation and Resource Utilization, Key Laboratory of Tea Biology and Tea Processing, Ministry of Agriculture and Rural Affairs, Anhui Agricultural University, Hefei, Anhui 230036, China; State Key Laboratory for Tea Plant Germplasm Innovation and Resource Utilization, Key Laboratory of Tea Biology and Tea Processing, Ministry of Agriculture and Rural Affairs, Anhui Agricultural University, Hefei, Anhui 230036, China; State Key Laboratory for Tea Plant Germplasm Innovation and Resource Utilization, Key Laboratory of Tea Biology and Tea Processing, Ministry of Agriculture and Rural Affairs, Anhui Agricultural University, Hefei, Anhui 230036, China; College of Life Science, Anhui Agricultural University, Hefei, Anhui 230036, China; State Key Laboratory for Development and Utilization of Forest Food Resources, Zhejiang A&F University, Hangzhou, Zhejiang 311300, China; State Key Laboratory for Tea Plant Germplasm Innovation and Resource Utilization, Key Laboratory of Tea Biology and Tea Processing, Ministry of Agriculture and Rural Affairs, Anhui Agricultural University, Hefei, Anhui 230036, China

**Keywords:** *Empoasca onukii*, elevational gradient, *Wolbachia*, cytoplasmic hitchhiking, insect microbiome, mitochondrial DNA

## Abstract

Mountain gradients act as natural experiments, and elevational mitochondrial DNA clines in insects are often interpreted as signatures of local metabolic adaptation. However, mitochondrial DNA is maternally co-inherited with heritable endosymbionts that can promote cytoplasmic hitchhiking and, when abundant, dominate marker-gene microbiome profiles, complicating inference about environmental forcing. Here, we evaluate a symbiont-aware cytoplasmic-axis framework in the tea green leafhopper *Empoasca onukii* using a densely replicated elevational survey across tea agroecosystems. Across 790 adults from 79 sites spanning 11 to 2750 meters above sea level, we quantified *Wolbachia* infection prevalence, within-host burden, and strain composition, and related these measures to mitochondrial haplotypes, a conservative nuclear reference marker, and whole-insect bacterial community profiles. *Wolbachia* prevalence, burden, and strain composition varied along elevation, with pronounced strain turnover. Mitochondrial diversity declined and haplotypes homogenized at high elevations, whereas the nuclear reference marker showed weak spatial structure, yielding mitochondrial–nuclear discordance consistent with cytoplasmic hitchhiking and sweep-like mtDNA homogenization. Bacterial community separation was strongest when *Wolbachia* features were retained but persisted after *Wolbachia* removal and renormalization, indicating both compositional dominance by *Wolbachia* and residual restructuring among non-*Wolbachia* taxa. In a balanced subset, mitochondrial coding variation and host energetic readouts provided observational functional context for the *Wolbachia*-centered cytoplasmic-axis pattern. Together, these results place *Wolbachia* at the center of a testable cytoplasmic framework linking elevational mitochondrial turnover with microbiome restructuring, and they highlight the broader importance of dominant heritable symbionts.

## Introduction

Mountain systems compress multiple abiotic stressors over short geographic distances, including low temperature, reduced oxygen availability, and elevated ultraviolet (UV) radiation, providing powerful “natural experiments” for insect adaptation [1]. These covarying gradients reshape insect physiology, life history, and population structure [2]. Accordingly, sharp elevational clines in mitochondrial DNA (mtDNA) are often interpreted as local selection on energy metabolism [3, 4]. Yet, elevation is a composite proxy rather than a single causal variable [5], and mtDNA is embedded within a cytoplasmic inheritance system [6].

In symbiont-bearing animals, mitochondria and cytoplasmic endosymbionts often share maternal transmission routes [7, 8]. Shifts in symbiont frequency—via drift, spatial spread, or selection acting on the symbiont—can therefore drive cytoplasmic hitchhiking, carrying linked mtDNA haplotypes to high frequencies [9, 10]. This can depress mtDNA diversity and produce a sweep-like structure that mimics local adaptation, even when mitochondrial function is not the primary target [11, 12]. A common expectation is that mtDNA structure can exceed differentiation at independently inherited nuclear loci, motivating cautious comparisons with nuclear markers that are not co-inherited through the cytoplasm [13]. Robust inference thus benefits from jointly evaluating symbiont dynamics, mtDNA variation, nuclear benchmarks, and host-associated microbial communities [14, 15]. In arthropods, *Wolbachia* is a pervasive, maternally inherited endosymbiont and often a dominant microbiome component [8, 16, 17], although non-vertical transmission has also been reported in some systems [18].

Microbiome data further help quantify a potentially dominant *Wolbachia* axis and assess whether elevational community differences—and associated mtDNA patterns—are dominated by *Wolbachia* compositional effects or extend to broader turnover among co-occurring taxa [14, 15]. Because *Wolbachia* can dominate whole-insect 16S rRNA gene profiles, apparent *α*-diversity and between-sample dissimilarities may track *Wolbachia* load; parallel analyses retaining versus removing *Wolbachia* can separate compositional dominance from residual restructuring [15, 19–22]. Beyond classical reproductive phenotypes, evidence—particularly from sap-feeding hemipterans—links *Wolbachia* to host nutrition and energy metabolism, making symbiont burden a plausible correlate of mitochondrial and microbiome variation [23–26]. At the same time, mitochondrially encoded oxidative phosphorylation (OXPHOS) genes can be genuine targets of abiotic selection, and focal amino-acid substitutions may modulate respiratory-chain performance under cold, low oxygen, or high-UV regimes [27, 28]. Thus, elevational mtDNA and microbiome patterns may reflect a symbiont-associated sweep background [10–12], mitochondrial functional variation, microbiome restructuring, or a combination of these processes under composite mountain stress.

The symbiont-aware framework is particularly relevant to typhlocybine leafhoppers (Typhlocybinae), where canonical obligate nutritional symbioses are not consistently retained and facultative associates—including *Wolbachia*—are often prevalent [29, 30]. We focus on the tea green leafhopper (TGLH), *Empoasca onukii*, a major pest of *Camellia sinensis* across Asian tea agroecosystems [31–33]. Tea landscapes provide replicated elevational mosaics that enable dense spatial replication and limit broad biome-level confounding [31, 32]. Genome-wide SNP analyses report pronounced regional structure in TGLH (often summarized as three major clusters, *K* ≈ 3), underscoring the need to consider host nuclear background; here, we use the 28S rRNA gene as a conservative nuclear reference marker independent of the cytoplasm [32, 33]. In addition, genome-based searches in TGLH did not recover evidence for a retained, canonical *Sulcia/Nasuia*-type obligate nutritional symbiosis, an inference consistent with comparative leafhopper-genome evidence [33, 34]. This apparent absence, together with previous metagenomic and field-based microbiome studies, supports the relevance of facultative and environmentally variable microbial partners, including *Wolbachia*, in TGLH [35, 36]. These features make TGLH a suitable system for distinguishing *Wolbachia* compositional dominance from residual restructuring among non-*Wolbachia* taxa.

Here, we evaluate whether elevational mtDNA turnover in TGLH can be interpreted within a *Wolbachia*-centered cytoplasmic framework that also captures variation in whole-insect bacterial communities. We characterize *Wolbachia* prevalence, within-host load, and strain composition across populations, and we compare mtDNA structure with a conservative nuclear reference marker. We then examine whether microbiome shifts persist after *Wolbachia* removal. To connect these layers, we integrate population-scale mtDNA, the 28S nuclear reference, targeted *ATP6/ATP8* variation, energetic readouts, and retained/depleted 16S rRNA gene profiles. Because elevation is a composite proxy, we evaluated climatically relevant variables, including ultraviolet A radiation (UVA), and use site-level structural equation modeling (SEM) to assess whether environment-linked mtDNA and microbiome patterns are statistically aligned with *Wolbachia* load.

## Materials and methods

### Field sampling and site metadata

From 2021 to 2023 (April–October), we sampled TGLH adults from 79 tea plantations across 17 provincial-level regions in China (*n* = 790; 19.15°–35.28°N; 11–2750 m a.s.l.; Table S1). Sites were grouped as low (<400 m), mid (400–1000 m), or high (≥1000 m) elevation. Near local peak abundance, 10 adults per site were randomly collected from tea bushes within a plantation or a contiguous tea-growing area of ~1–2 km, with bushes spaced 20–50 m apart, preserved in 95%–99% ethanol, and stored at −20°C until DNA extraction. WGS84 coordinates, elevation, sampling date, and site description were recorded for each site.

### Environmental characterization

We combined GPS-recorded elevation with site-level climatic variables from NASA POWER [37]. Daily data were extracted from the nearest grid cell and summarized over 2019–2023 as multi-year site-level metrics. Total annual precipitation was calculated as an annual total, whereas ultraviolet radiation, temperature, relative humidity, surface pressure, and temperature-range variables were summarized as annual means. The evaluated variables included elevation, surface pressure (P), mean temperature (T), relative humidity (RH), mean temperature range (MTR), extreme temperature range (ETR), total annual precipitation (TAP), ultraviolet A radiation (UVA), and ultraviolet B radiation (UVB). The names, units, and summaries of all evaluated variables are provided in Table S1.

### DNA extraction and host species identification

Adults were surface-sterilized (75% ethanol, 5 min), rinsed three times in sterile water, and air-dried. Whole-body DNA was extracted using DNeasy Blood & Tissue Kit (Qiagen). Extraction blanks were included during DNA extraction, and DNA quality/quantity were assessed (gel electrophoresis, NanoDrop, Qubit); low-quality samples were re-extracted with randomized batch order.

Species identity (*n* = 790) was confirmed by amplifying the 28S rRNA gene (D8 region) [38] and mitochondrial *COI/COII* [39] (primers and cycling in Table S2), followed by bidirectional Sanger sequencing. Reads were aligned with MAFFT [40] and edited in MEGA11/Geneious [41, 42]. Assignments were verified by BLASTn [43] against curated BOLD references [44] (*COI* ≥ 98.5% identity and ≥ 95% coverage), requiring concordance across *COII* and *28S*; all retained samples met these criteria.

### *Wolbachia* detection, quantification, and strain typing

*Wolbachia* infection was screened by *wsp* PCR (81F/691R; Table S2) [45] with positive and negative controls (extraction blanks, no-template controls, and uninfected insects). Samples with weak/ambiguous bands were re-amplified, with negative controls carried through the same steps, and only reproducible bands obtained with clean negative controls were used for final scoring. All reproducible *wsp*-positive amplicons were Sanger-sequenced and BLAST-verified. Individuals were scored as infected (WI) if the expected amplicon was reproducibly obtained; otherwise, they were scored as uninfected (WU).

*Wolbachia* relative titer in WI individuals was estimated semi-quantitatively by *ftsZ* qPCR [25] normalized to host *β-actin* (Table S2) [46]. For each sample, *ftsZ* and *β-actin* were measured with three technical replicates, and mean Cq values were used. Relative *Wolbachia* burden was estimated from ΔCt = Ct(*ftsZ*) − Ct(*β-actin*). For continuous analyses, Ct(*ftsZ*) = 35 was used as the low-burden reference threshold in the 2^−ΔΔCt^ calculation [47]; values with Ct(*ftsZ*) ≥ 35 were considered beyond the reliable continuous quantification range. Titers were log_2_-transformed for downstream models. qPCR-derived values were used only as semi-quantitative estimates of relative *Wolbachia* burden and not for infection calling.

Strains were typed from *wsp*-positive individuals using MLST (*gatB, coxA, hcpA, ftsZ, fbpA*) plus *wsp* (Table S2) [48], with allele calls and ST assignment via PubMLST/BIGSdb [49]. Samples that were *wsp*-positive but lacked complete MLST profiles were retained as infected but treated as untyped in strain-level analyses. Unique *wsp* haplotypes were placed in a maximum-likelihood tree with A/B supergroup reference panels; reference accessions and panel composition are provided in Table S3.

### Mitochondrial variation, nuclear reference, and population genetic analyses

We analyzed mitochondrial *COI/COII* together with the nuclear 28S rRNA gene (D8 region) as a conservative nuclear reference marker [38]. *COI/COII* sequences were BLAST-validated, aligned with MAFFT [40], collapsed to haplotypes, and numbered by descending frequency. The concatenated *COI* + *COII* fragment (~1370 bp) was used for full-dataset mtDNA summaries (790 individuals from 79 populations) and, after excluding mid-elevation sites (400–1000 m), for reduced low/high endpoint analyses (650 individuals from 65 populations). Diversity and neutrality statistics, including the number of haplotypes (Nhap), haplotype diversity (Hd), nucleotide diversity (π), Tajima’s D, and Fu’s Fs, were calculated in DnaSP [50]. Haplotype relationships were visualized as TCS networks in POPART [51], and structure was assessed using analysis of molecular variance (AMOVA) and pairwise fixation index (*F*_ST_) in Arlequin (10 000 permutations) [52]. Mismatch distributions, including *τ*, the sum of squared deviations (SSD), and Harpending’s raggedness, were evaluated with 10 000 bootstrap replicates, and sliding-window π was calculated in DnaSP using a 100-bp window and a 25-bp step. Evenness/dominance metrics (Shannon H′ and Berger–Parker *p*_max_) were calculated in R (vegan) [53]. To account for unequal sampling among elevation-by-infection groups, Hd and π were additionally assessed by rarefaction via without-replacement subsampling to *n* = 60 individuals per elevation × infection-status group (600 iterations).

### *ATP6/ATP8* sequencing and selection analyses

*ATP6* and *ATP8* were sequenced from a balanced subset of 80 adults selected to balance across elevation (high/low) and infection status (WI/WU), yielding four groups (WIHigh, WILow, WUHigh, WULow; *n* = 20 per group; Table S4). Major *COI* + *COII* haplotype backgrounds and multiple population sources were retained within this subset. Primers were designed based on the TGLH mitogenome (Table S2). After alignment, *ATP6/ATP8* coding regions were extracted, translated, and amino-acid positions were indexed to the TGLH mitogenome. Genetic diversity and neutrality tests were performed in DnaSP, and between-group differentiation (*F*_ST_) was assessed in Arlequin. Nonsynonymous variants were screened, and amino-acid differences among groups were tested using two-sided Fisher’s exact tests with Benjamini–Hochberg correction [54]. Conservation around focal sites was summarized with WebLogo [55]. Structural context was summarized using AlphaFold predictions [56], putative functional effects were predicted with SIFT and PROVEAN [57, 58], codon-level selection was assessed using EasyCodeML [59], and structural visualizations were generated in PyMOL [60].

### ATP quantification and enzyme activity assays

ATP content, Complex V activity, and citrate synthase (CS) activity were compared between Pop16 and Pop49, representing high- and low-elevation populations with contrasting *Wolbachia* prevalence/titer profiles (Table S1). Adults were flash-frozen in liquid nitrogen in the field, stored at −80°C (≤1 freeze–thaw before assays), and processed on ice. Each biological replicate pooled 100 adults of similar size, with eight biological replicates per population per assay. Because assay buffers were incompatible, ATP, Complex V, and CS were measured from independently prepared parallel pools; ATP and Complex V were reported per 100 adults and CS as U·g^−1^ tissue. ATP was quantified using a commercial micromethod kit. Complex V activity was measured as oligomycin-sensitive ATP hydrolysis at 340 nm, and CS activity by the 5,5′-dithiobis (2-nitrobenzoic acid) (DTNB) method at 412 nm; assays were run on microplates with standards and three technical replicates per biological replicate, and over-range samples were diluted and re-read.

### Microbiome profiling and analyses

Whole-insect bacterial communities were profiled by 16S rRNA gene amplicon sequencing from the same 80 individuals used for *ATP6/ATP8* analyses (WIHigh, WILow, WUHigh, WULow; *n* = 20 each; Table S4). The V3–V4 region of the bacterial 16S rRNA gene was amplified with 341F/806R and sequenced on a NovaSeq 6000 System (Illumina) with paired-end reads. Paired-end reads were demultiplexed by barcode, merged, quality-filtered, screened for chimeras, denoised into amplicon sequence variants (ASVs) using QIIME2/DADA2, and taxonomically assigned against the SILVA 138.1 reference database. Downstream statistical analyses were then performed using R-based workflows. We removed chloroplast and mitochondrial ASVs, non-bacterial ASVs, and extremely low-abundance ASVs (<0.01% total relative abundance), excluded low-quality libraries, and retained the resulting filtered bacterial ASV table for downstream analyses. Filtered features were renormalized to relative abundances where required for community-composition and abundance-based analyses. Sequencing-depth coverage was evaluated using observed-ASV rarefaction curves for both *Wolbachia*-retained and *Wolbachia*-depleted tables (Fig. S1). Metadata, read counts before and after filtering, *Wolbachia* relative abundance estimated from 16S rRNA gene amplicon data, and filtered ASV tables are provided in DataSheets S1–S2.

To distinguish compositional dominance by *Wolbachia* from residual community restructuring, we analyzed two parallel tables: *Wolbachia*-retained (all filtered bacterial ASVs) and *Wolbachia*-depleted (*Wolbachia* ASVs removed, then renormalized). Alpha diversity, including the Shannon Index and observed ASVs, was computed on rarefied tables and compared among groups using Kruskal–Wallis tests with Benjamini–Hochberg correction for post hoc comparisons. Beta diversity was assessed using Bray–Curtis dissimilarities and visualized by principal coordinates analysis (PCoA); group differences were tested using permutational multivariate analysis of variance (PERMANOVA; adonis2; 9999 permutations) [61] and checked with permutational analysis of multivariate dispersions (PERMDISP; betadisper) [62]. In the retained table, *Wolbachia* relative abundance was compared among groups, and among *wsp*-positive individuals we assessed concordance between *Wolbachia* relative abundance estimated from 16S rRNA gene amplicon data and qPCR-derived relative titer using Spearman rank correlation.

Genus-level co-abundance networks were inferred from the *Wolbachia*-depleted table using FastSpar [63], consistent with compositional data principles [64]. ASVs were agglomerated to genus level and genera present in ≥10% of samples were retained; correlations were bootstrapped and filtered using standard effect-size and multiple-testing criteria [54]. Modules were detected using Louvain community detection (igraph) [65], per-sample module abundance was defined as the summed relative abundance of member genera, and module differences between WIHigh and WILow were tested using Welch’s *t*-tests with Benjamini–Hochberg correction; module abundance was related to qPCR titer using Pearson correlation and linear regression. Exploratory functional profiles were inferred from *Wolbachia*-depleted 16S rRNA gene amplicon data using PICRUSt2 to predict KEGG Orthologues (KOs) [66], and differential KOs were tested under a centered log-ratio framework with multiple-testing correction [54, 64]. In parallel, six pooled whole-insect shotgun metagenomes generated and reported in our previous study [35] from an independent high- versus low-elevation contrast (High *n* = 3 pools, Low *n* = 3 pools) were re-analyzed here as an independent metagenomic background comparison and interpreted qualitatively given the pooled design and limited group size.

### Structural equation modeling

To assess whether elevation- or UVA-associated variation in mtDNA diversity and microbiome structure was statistically aligned with *Wolbachia* load, we fitted site-level SEMs after excluding mid-elevation sites (400–1000 m; *n* = 65 populations). Variables were z-standardized. Two models with identical topology were fitted, using either elevation (m a.s.l.) or long-term mean UVA (NASA POWER, 2019–2023) [37] as the exogenous driver. *Wolbachia* load was modeled as a latent factor indicated by site infection prevalence (%) and site-level mean log_2_-transformed qPCR-derived relative titer [47]. Outcomes were (i) a latent mtDNA diversity factor indicated by *COI* + *COII* Hd and π, and (ii) microbiome structure summarized by PCoA1 from Bray–Curtis dissimilarities calculated from the *Wolbachia*-depleted ASV table. Models were fitted in R (lavaan) [67] using robust maximum likelihood (MLR) with full-information maximum likelihood (FIML) for missingness; indirect path estimates were assessed using 5000 bias-corrected bootstrap resamples. We report standardized path coefficients, indirect effects with 95% confidence intervals, *R*^2^, and global fit indices, including comparative fit index (CFI), root mean square error of approximation (RMSEA), and standardized root mean square residual (SRMR).

To account for differences in analytical scale among population-level mtDNA summaries, the balanced 80-individual 16S rRNA gene amplicon subset, and infected-only comparisons between qPCR-derived relative titer and 16S rRNA gene amplicon-based *Wolbachia* relative abundance, we also performed supporting scale-specific analyses. These included population-level mtDNA models, dissimilarity-based microbiome models for the *Wolbachia*-retained and *Wolbachia*-depleted 16S rRNA gene tables, and infected-only Spearman correlation/regression between qPCR-derived relative titer and *Wolbachia* relative abundance estimated from 16S rRNA gene amplicon data.

### Statistical analyses

All analyses were performed in R v4.2.2. Continuous predictors were z-standardized unless otherwise stated, and two-sided *α* = 0.05 was used. Group contrasts used nonparametric tests when assumptions were not met, and multiple testing was controlled using Benjamini–Hochberg correction. *Wolbachia* prevalence was modeled at the site level using a binomial generalized linear model (GLM) with a logit link infected vs uninfected counts per site, and model performance was summarized using Tjur’s *R*^2^; elevation was scaled to 100-m units to report the odds ratio per 100 m increase in elevation (OR_100m_) with 95% confidence intervals. Titer analyses were restricted to *wsp*-positive individuals and modeled using weighted site-level linear models based on mean log₂-transformed qPCR-derived relative titer, with the number of individuals contributing qPCR-based titer estimates used as weights. A quadratic elevation term was included for titer analyses when supported by model diagnostics and the Akaike information criterion (AIC).

Elevation-only, UVA-only, and elevation + UVA focal models were compared using the same response definitions. Multicollinearity among the evaluated environmental variables was assessed using variance inflation factors (VIFs) to document covariance and guide interpretation, rather than as an automatic variable-retention procedure. Detailed coefficients, *P* values, AIC, ΔAIC, sample sizes, *R*^2^ values, and VIF outputs are provided in DataSheet S3.

## Results

### Elevation-linked variation in *Wolbachia* prevalence, burden, and strain composition

**Figure 1:**
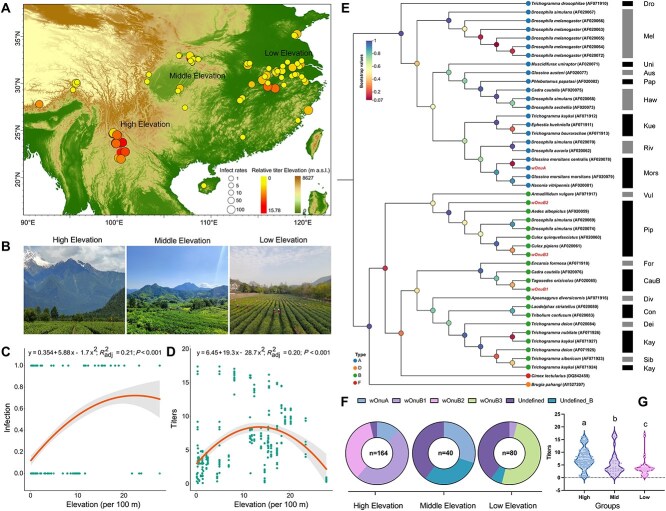
Elevation-linked structuring of *Wolbachia* prevalence, within-host titer, and strain composition. (A) Sampling map of 79 tea plantations across China (11–2750 m a.s.l.). Elevation zones are defined as low (<400 m), mid (400–1000 m), and high (≥1000 m). Point size indicates site-level *Wolbachia* infection prevalence, defined as the percentage of *wsp*-positive individuals within each population, and point color indicates site-level mean within-host *Wolbachia* relative titer among infected individuals at each site. Relative titer was estimated by *ftsZ* qPCR normalized to host *β-actin* and expressed on a log₂ scale. (B) Representative tea-plantation sites along the elevation gradient. (C) Individual-level *Wolbachia* infection status (0/1) versus elevation, based on *wsp* PCR screening of 790 individuals from 79 populations. The fitted curve is from a site-level binomial GLM using infected and uninfected counts per population, with elevation expressed per 100 m and including a quadratic elevation term; the model equation and significance are shown on the panel. (D) Relationship between within-host *Wolbachia* relative titer and elevation among infected individuals (*n* = 284), with a quadratic fit; the fitted peak occurs at intermediate elevation as indicated by the panel equation. (E) Maximum-likelihood phylogeny of *wsp* sequences showing supergroup placements and focal strains, including *w*OnuA and *w*OnuB1–B3. (F) *Wolbachia* strain/typing-category composition among *wsp*-positive individuals. Pie charts summarize strain composition within high, mid, and low elevation zones, with sample sizes shown in the pies. Undefined indicates *wsp*-positive individuals without complete five-locus MLST profiles, and Undefined_B indicates *wsp*-positive infections assigned to supergroup B but lacking complete profiles for strain-level assignment. (G) Within-host *Wolbachia* relative titer across elevation zones among infected individuals; sample sizes are high *n* = 164, mid *n* = 40, and low *n* = 80. Different letters indicate significant differences among elevation zones after multiple-testing correction.

We screened 790 adult TGLH from 79 tea plantations spanning 11–2750 m a.s.l. (Fig. 1A and B; Table S1). *Wolbachia* was detected by *wsp* PCR in 284/790 individuals (35.9%) across 37/79 populations (46.8%; Table S5; DataSheet S1). Infection was detected in 15 of 42 low-elevation populations, 5 of 14 mid-elevation populations, and 17 of 23 high-elevation populations, with mean site-level prevalences of 19.0%, 28.6%, and 71.3%, respectively. Infection prevalence increased along the elevational gradient in site-level binomial models, with the elevation-only model estimating OR_100m_ = 1.16 (95% CI, 1.13–1.19; *P* < .001) and the linear + quadratic elevation model supporting a nonlinear elevational pattern (*P* < .001; Fig. 1C; DataSheet S3).

Among *wsp*-positive individuals, within-host *Wolbachia* relative titer varied nonlinearly with elevation and reached a model-predicted peak at ~1357 m (adjusted *R*^2^ = 0.21; *P* < .001; Fig. 1D). Across elevation zones, *Wolbachia* relative titer was highest at ≥1000 m (*n* = 164; 7.55 ± 3.97), intermediate at 400–1000 m (*n* = 40; 5.22 ± 4.22), and lowest at <400 m (*n* = 80; 3.69 ± 3.09; mean ± SD, log_2_ scale; Fig. 1G; DataSheet S1). Individual-level titer showed substantial within-population variation across infected populations from all three elevation zones (Fig. S2).

In site-level environmental screening models, UVA showed the strongest single-predictor performance for infection prevalence (Tjur’s *R*^2^ = 0.39), whereas UVA and UVB showed similar performance for site-level mean relative titer (weighted *R*^2^ = 0.12 and 0.13, respectively) (Fig. S3A and B; DataSheet S3). In focal comparisons, the UVA-only model outperformed the elevation-only model for infection prevalence (Tjur’s *R*^2^ = 0.39 vs 0.21) and modestly improved titer fit (weighted *R*^2^ = 0.12 vs 0.09) (Fig. S3C and D; DataSheet S3).

To determine whether the elevation-linked infection and burden patterns were accompanied by strain turnover, we typed *Wolbachia* in *wsp*-positive individuals with complete five-locus MLST profiles (*n* = 218). Four strains were identified: *w*OnuB1, *w*OnuB2, *w*OnuB3, and *w*OnuA, hereafter B1, B2, B3, and A. Supergroup assignments were concordant between *wsp* phylogeny and MLST loci (Fig. 1E; Table 1; Table S5). Among successfully typed infections, strain composition was clearly structured by elevation: high-elevation populations were dominated by B1/B2 strains, whereas low-elevation typed infections were dominated by B3, with B2 restricted to high-elevation sites and A detected mainly at intermediate–high elevations (Fig. 1F; Table 1).

**Table 1:** Summary of *Wolbachia* strains inferred from MLST typing in the tea green leafhopper (TGLH).

Strain	Supergroup	MLST allelic profile^a^	Typed individuals	Typed populations	Elevation range (m a.s.l.)
*w*OnuB1 (B1)	B	105–14–13-7-317	84	9	100–2034
*w*OnuB2 (B2)	B	158–88–13-105-302	58	6	1169–2750
*w*OnuB3 (B3)	B	158–88–142-7-317	50	5	30–820
*w*OnuA (A)	A	58–48–60-14-16	26	3	700–1740

^a^The MLST allelic profile is reported for the five loci in the order *gatB–coxA–hcpA–ftsZ–fbpA*. No. typed individuals and No. typed populations indicate the numbers of *wsp*-screening-positive individuals and populations with complete five-locus MLST sequence profiles assigned to each strain. Supergroup assignment was based on MLST loci and cross-validated using the *wsp* phylogeny. Because some complete allelic profiles did not correspond to an existing sequence type in PubMLST, strain labels are reported here together with their allelic profiles. Full site-level typing information, including incomplete locus sets and PubMLST-unassigned profiles, is provided in Table S5 and DataSheet S1.

### *Wolbachia* infection coincides with reduced mtDNA diversity and Hap1 dominance at high elevation

**Figure 2:**
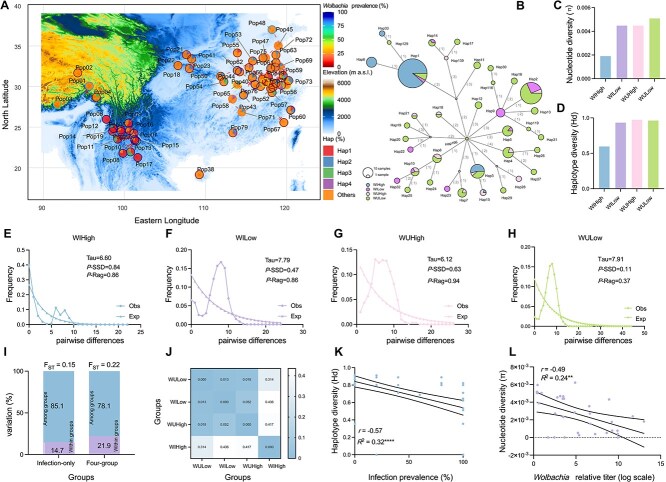
WIHigh samples show reduced mitochondrial diversity and Hap1 dominance. (A) Spatial distribution of common concatenated *COI* + *COII* haplotypes across 65 low- and high-elevation populations after excluding mid-elevation sites (400–1000 m); pie charts show Hap1–Hap4 with remaining haplotypes pooled, and circle outlines indicate site-level *Wolbachia* prevalence. (B) TCS network of common *COI* + *COII* haplotypes (≥4 individuals); node size is proportional to haplotype frequency and pie charts indicate contributions from four groups: WIHigh (infected, high elevation), WILow (infected, low elevation), WUHigh (uninfected, high elevation), and WULow (uninfected, low elevation). Numbers along branches indicate mutational steps. (C–D) Nucleotide diversity (π) and haplotype diversity (Hd) of *COI* + *COII* by group. (E–H) Mismatch distributions for each group, with τ, SSD, and Harpending’s raggedness statistics shown in each panel. (I) AMOVA variance partitioning under infection-only grouping and four-groups grouping. Bars show the percentages of variation explained among and within groups. (J) Pairwise *F*st among groups. (K) Population-level association between *Wolbachia* prevalence and Hd. (L) Population-level correlation between *Wolbachia* titer and π. lines in (K) and (L) indicate fitted linear regressions with 95% confidence intervals.

To minimize ecological heterogeneity, we excluded mid-elevation sites (400–1000 m) and analyzed 650 adults from 65 low- and high-elevation populations using concatenated *COI* + *COII* and nuclear 28S rRNA gene as a conservative reference marker, stratified by infection status and elevation (WIHigh, *n* = 164; WUHigh, *n* = 66; WILow, *n* = 80; WULow, *n* = 340). The endpoint *COI* + *COII* dataset comprised 178 haplotypes, whereas the full-gradient dataset comprised 190 haplotypes (Table S6), with clear spatial variation in the dominant haplotypes (Hap1–Hap4; Fig. 2A; Fig. S4).

Mitochondrial diversity differed strongly among groups (Fig. 2C and D; Table S6). Infected high-elevation samples (WIHigh) showed the lowest diversity (Hd = 0.600, π = 0.00203), whereas uninfected high-elevation samples maintained substantially higher diversity (WUHigh: Hd = 0.976, π = 0.00479), with WILow and WULow also remaining high (Hd = 0.932 and 0.965; π = 0.00499 and 0.00545, respectively; Table S6). The same pattern was retained in the full-gradient summary and rarefaction analysis (Table S6; Fig. S5). Sliding-window analysis showed reduced nucleotide diversity across much of the concatenated *COI* + *COII* region in WIHigh (Fig. S6). Haplotype relationships further revealed pronounced centralization around Hap1 (Fig. 2B; full network in Fig. S4), which accounted for 62.2% of WIHigh sequences but was rare or absent in the other groups (WILow 5.0%, WUHigh 0.0%, WULow 3.2%), accompanied by reduced evenness and increased dominance in WIHigh (Fig. S4).

Multiple complementary analyses supported the same pattern (Figs 2E–J, S5, and  S6; Table S6), including AMOVA (14.7% variance explained by infection status grouping; 21.9% by the four-group partition; Fig. 2I) and elevated differentiation in contrasts involving WIHigh (e.g. WUHigh vs WIHigh *F*_ST_ = 0.42; WIHigh vs WILow *F*_ST_ = 0.44; Fig. 2J). At the population level, infection prevalence was negatively associated with Hd (*r* = −0.57, *R*^2^ = 0.32, *P* < .001; Fig. 2K) and site-level mean qPCR-derived relative *Wolbachia* titer was negatively associated with π (*r* = −0.49, *R*^2^ = 0.24, *P* = .004; Fig. 2L). In contrast, 28S showed low diversity and minimal differentiation (Table S6).

### *ATP6* L139I is enriched at high elevation with higher Complex V activity observed in representative population pools

**Figure 3:**
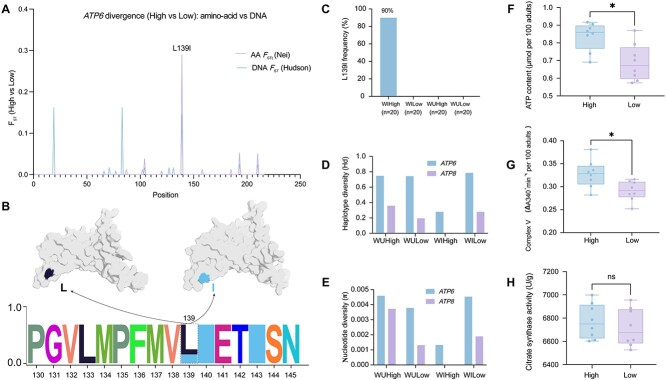
*ATP6* L139I and elevation-associated differences in ATP content and Complex V activity. (A) Per-site *F*_ST_ (high vs low) scan across *ATP6* at both the amino-acid and nucleotide levels; codon 139 shows a concordant peak corresponding to the nonsynonymous substitution L139I. (B) Predicted structural context and local sequence logo (aa130–145) highlighting position 139. (C) Frequency of the *ATP6* 139I allele across elevation (pooled) and across the four groups (*n* = 20 per group). (D, E) *ATP6* and *ATP8* diversity across groups: Haplotype diversity (Hd; D) and nucleotide diversity (π; E). (F) Whole-animal ATP content and (G) oligomycin-sensitive Complex V activity in representative high- versus low-elevation populations (Pop16 versus Pop49; *n* = 8 pools per population; 100 adults per pool). (H) Citrate synthase (CS) activity for the same comparison. Asterisks indicate significant differences between representative high- and low-elevation population pools; ns indicates not significant.

Guided by the mtDNA grouping pattern, we sequenced mitochondrial ATP synthase genes in a four-group design (WUHigh, WULow, WIHigh, WILow; *n* = 20 per group). A joint amino-acid/DNA *F*_ST_ scan across *ATP6* revealed a single concordant divergence peak at codon 139 (L139I), whereas other sites were near baseline (Fig. 3A). Residue 139 mapped to a predicted transmembrane segment and was the primary variable site in the local sequence context (Fig. 3B).

When pooled by elevation (*n* = 40 per elevation), the I variant reached 45.0% at high elevation and was absent at low elevation (0%; Fisher’s exact test, *P* < .001; Fig. 3C); at the group level it was concentrated in WIHigh (90%; Fig. 3C). Consistent with this skew, *ATP6* diversity was lowest in WIHigh (Hd = 0.28; π = 0.00132) compared with WUHigh (Hd = 0.75; π = 0.00459) and WILow (Hd = 0.78; π = 0.00453), with WULow intermediate (Hd = 0.74; π = 0.00378) (Fig. 3D and E).

For *ATP8*, DNA-level diversity decreased at high elevation, whereas amino-acid variation remained limited (I17T in 2/80 sequences; *P* = .664; Fig. 3D and E; Fig. S7). In physiological assays on representative high- and low-elevation population pools (100 adults per pool; *n* = 8 pools per population), ATP content and oligomycin-sensitive Complex V activity were higher in the high-elevation pools than in the low-elevation pools (Fig. 3F and G): ATP, 0.86 versus 0.67 μmol per 100 adults, 1.28-fold higher (Mann–Whitney U = 9, exact *P* = .015); Complex V activity, 0.329 vs 0.292 ΔA340·min^−1^ per 100 adults, 1.13-fold higher (U = 10, exact *P* = .021). Citrate synthase activity did not differ (High: 6749; Low: 6675 U g^−1^; U = 25, *P* = .505) (Fig. 3H). Codon-model analyses did not identify Bayes empirical Bayes (BEB)-supported sites under positive selection in ATP6 (Table S7).

### *Wolbachia*-retained and *Wolbachia*-depleted 16S rRNA gene profiles differ among groups.

**Figure 4:**
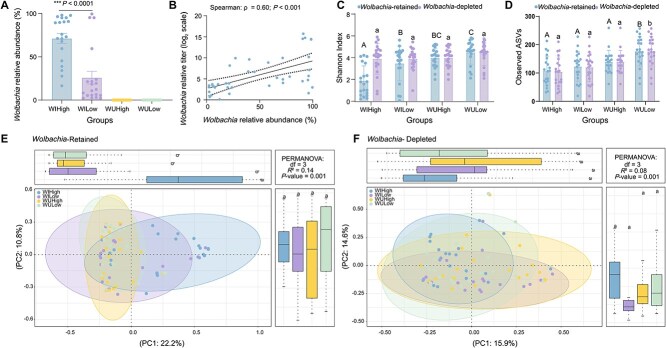
*Wolbachia* dominates bacterial community composition and contributes strongly to *β*-diversity separation. The same 80 individuals used for *ATP6/ATP8* analyses were profiled by 16S rRNA gene amplicon sequencing, with *n* = 20 per group. (A) *Wolbachia* relative abundance (%) across groups WIHigh, WILow, WUHigh, and WULow in the *Wolbachia*-retained ASV table. (B) Relationship between *Wolbachia* relative abundance estimated from 16S rRNA gene amplicon data and log₂-transformed qPCR-derived relative titer among infected individuals. Spearman correlation is shown. (C–D) Alpha diversity (Shannon Index, C; and Observed ASVs, D) with *Wolbachia* retained and after *Wolbachia* ASVs were removed followed by renormalization. Uppercase and lowercase letters indicate significant differences among groups within the retained and depleted tables, respectively. (E–F) Bray–Curtis PCoA of the *Wolbachia*-retained and *Wolbachia*-depleted ASV tables; PERMANOVA statistics are shown in each panel. Ellipses indicate group dispersion, and marginal boxplots show the distributions of PCoA1 and PCoA2 scores for each group. PERMDISP results are provided in Fig. S8.

We profiled whole-insect bacterial communities from the same 80 adults used for *ATP6/ATP8* analyses across four groups: WIHigh, WILow, WUHigh, and WULow (*n* = 20 each). In *Wolbachia*-retained profiles, *Wolbachia* dominated infected individuals and was higher in WIHigh than WILow (median 74.6% vs 11.6%; Mann–Whitney U = 335, *P* < .0001), whereas both uninfected groups were near zero (Fig. 4A). Among infected individuals, *Wolbachia* relative abundance estimated from 16S rRNA gene amplicon data tracked qPCR-derived relative titer (Spearman *ρ* = 0.60, *P* < .001; Fig. 4B). Alpha diversity differed between the *Wolbachia*-retained and *Wolbachia*-depleted tables (Fig. 4C and D).

Bray–Curtis PCoA showed a larger group effect in the *Wolbachia*-retained table than in the *Wolbachia*-depleted table (PERMANOVA: retained table, df = 3, *R*^2^ = 0.14, *P* = .001; depleted table, df = 3, *R*^2^ = 0.08, *P* = .001; Fig. 4E and F). Non-metric multidimensional scaling (NMDS) ordinations based on the same Bray–Curtis dissimilarities showed consistent overall patterns, and PERMDISP did not detect significant differences in multivariate dispersion in either table (Fig. S8). Pairwise contrasts were strongest for WIHigh in the retained table, especially against WUHigh and WULow (*R*^2^ = 0.23–0.24; BH-adjusted *P* = .003; Table S8). After *Wolbachia* removal, WIHigh remained separated from the other groups, but with smaller effect sizes (*R*^2^ ≈ 0.07; BH-adjusted *P* ≤ .006; Table S8). A phylogeny-resolved view of *Wolbachia*-depleted ASVs suggested broadly shared lineages across groups with shifts primarily in relative abundance (Fig. S9).

**Figure 5:**
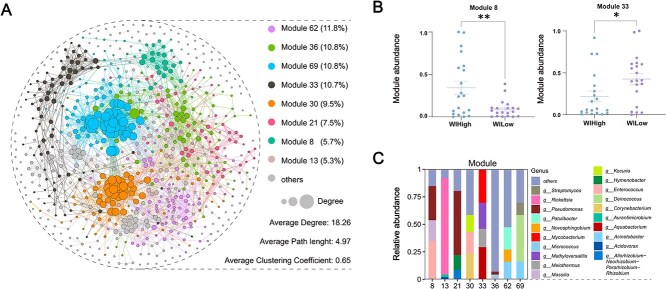
*Wolbachia*-depleted co-abundance network identifies non-*Wolbachia* modules differentiating infected high- and low-elevation individuals. (A) Genus-level co-abundance network inferred from the *Wolbachia*-depleted ASV table. Node color indicates module assignment, and node size indicates degree. The dominant modules and network-level statistics are shown in the panel. (B) Relative abundance of the two differentially abundant modules, Module 8 and Module 33, in WIHigh and WILow individuals. Module abundance was calculated as the summed relative abundance of member genera. Differences between WIHigh and WILow were tested using Welch’s t-tests with Benjamini–Hochberg correction. (C) Genus-level composition of Module 8 and Module 33. Stacked bars show the relative contribution of bacterial genera within each module; low-abundance genera are grouped as “others”.

Co-abundance analysis of the *Wolbachia*-depleted table further resolved this constrained residual variation (Fig. 5A), with Module 8 dominated by *Pseudomonas* (Fig. 5C). Among infected individuals, only two modules differed between WIHigh and WILow (Module 33 higher in WILow, *P* < .05; Module 8 higher in WIHigh, *P* < .01; Fig. 5B; full module comparisons in Fig. S10), and module–titer associations were modest (Module 33: *r* = −0.38, *P* < .05; Module 8: *r* = 0.20, *P* < .05; Fig. S11). Exploratory functional profiles suggested amino-acid-related signals in *Wolbachia*-depleted communities and pooled metagenomic backgrounds (Fig. S12).

### Structural equation models support statistical alignment among environment, *Wolbachia* load, mtDNA diversity, and microbiome structure

**Figure 6:**
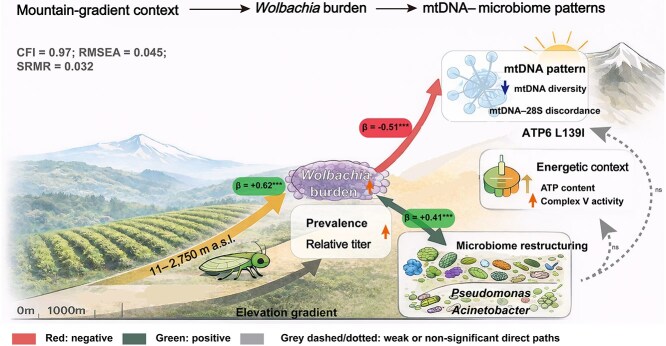
Site-level structural equation model summarizes statistical alignment among elevation, *Wolbachia* burden, mtDNA diversity, and microbiome structure. Site-level SEM with elevation as the exogenous driver. *Wolbachia* burden is modeled as a latent factor indicated by infection prevalence and site-level mean qPCR-derived relative titer. Outcomes include (i) a latent mtDNA diversity factor indicated by Hd and π and (ii) microbiome structure summarized by PCoA1 from Bray–Curtis dissimilarities calculated from the *Wolbachia*-depleted ASV table. Standardized path coefficients and model fit indices (CFI, RMSEA, SRMR) are shown. Grey dashed arrows denote tested direct paths that were weak or non-significant (ns).

We fitted site-level SEMs in which *Wolbachia* load was modeled as a latent factor indicated by infection prevalence and within-host titer (Fig. 6; Table S9). In the elevation model, elevation was positively associated with *Wolbachia* load (*β* = 0.62, *P* < .001; *R*^2^ = 0.38). *Wolbachia* load was associated with lower mtDNA diversity (*β* = −0.51, *P* < .001; *R*^2^ = 0.26) and shifts in the *Wolbachia*-depleted microbiome (PCoA1; *β* = 0.41, *P* < .001; *R*^2^ = 0.17). The direct paths from elevation to mtDNA diversity and to microbiome PCoA1 were not significant, whereas indirect path estimates via *Wolbachia* load were significant for both outcomes (mtDNA: *β*_ind_ = −0.32, 95% CI −0.47 to −0.19, *P* < .001; microbiome: *β*_ind_ = 0.26, 95% CI 0.12 to 0.41, *P* < .001).

Replacing elevation with long-term mean UVA increased the association between the environmental driver and *Wolbachia* load (*β* = 0.71, *P* < .001; *R*^2^ = 0.50) and yielded comparable indirect effects (mtDNA: *β*_ind_ = −0.36, 95% CI −0.51 to −0.23; microbiome: *β*_ind_ = 0.29, 95% CI 0.15 to 0.44; both *P* < .001). A small residual direct association between UVA and microbiome structure remained (*β* = 0.15, *P* = .041), and variance explained for microbiome structure increased (*R*^2^ = 0.28). Model fit was acceptable for both specifications and slightly improved under UVA (CFI = 0.98 vs 0.97; RMSEA = 0.035 vs 0.045; SRMR = 0.028 vs 0.032) (Fig. 6; Table S9).

Supporting scale-specific analyses showed concordant associations among mountain-gradient variables, *Wolbachia* load/abundance, mtDNA diversity, and microbiome structure in both *Wolbachia*-retained and *Wolbachia*-depleted profiles, including a negative association between composite *Wolbachia* load and mtDNA diversity and concordance between *Wolbachia* relative abundance estimated from 16S rRNA gene amplicon data and qPCR-derived relative titer (Table S10).

## Discussion

Mountain gradients compress cold, reduced oxygen availability, and ultraviolet radiation over short distances. In insects, sharp elevational clines in mtDNA are therefore often taken as evidence for local metabolic adaptation [1–5]. Our data point to a complementary, more cautious reading in a symbiont-rich host: in TGLH, variation in *Wolbachia* prevalence, within-host burden, and strain composition covaries with mtDNA turnover and whole-insect bacterial community structure. Across a densely replicated transect, *Wolbachia* prevalence, within-host burden, and strain composition were clearly structured along elevation, and infected high-elevation populations showed a marked contraction of mitochondrial barcode diversity and haplotype dominance.

A key clue comes from the nuclear reference marker. Mitochondria are not independent markers in hosts carrying maternally inherited symbionts [7, 8, 10, 13]. Relative to mtDNA, the independently inherited nuclear locus showed weak spatial structure, providing a conservative mito–nuclear reference compatible with cytoplasmic hitchhiking [6–13]. Taken together, these patterns are consistent with a *Wolbachia*-centered cytoplasmic process contributing to sweep-like mtDNA patterns and shaping 16S rRNA gene community profiles when a single intracellular taxon becomes abundant.

The cytoplasmic-hitchhiking interpretation complements previous mtDNA-based population and landscape-genetic studies of TGLH [31, 68], which linked mitochondrial structure to geographic distance, environmental heterogeneity, and landscape resistance. Using partly overlapping mtDNA markers, we found that the strongest homogenization was not shared by all high-elevation samples but was concentrated in the *Wolbachia*-infected high-elevation cytoplasmic background, with Hap1 dominant in WIHigh but absent from WUHigh. Thus, the mtDNA pattern should not be read only as a geographic or high-elevation signal, but also in relation to the maternally associated *Wolbachia* background.

Here, ultraviolet indices—especially long-term mean UVA—were the most consistent correlates of both infection prevalence and within-host burden, and UVA-based specifications performed better than elevation-based models in the site-level SEM framework. We do not treat this as proof that UVA alone drives the pattern; instead, it identifies a concrete environmental axis that was more closely aligned with *Wolbachia* variation than elevation alone in our models. Our ATP synthase results fit a mixed picture. *ATP6* divergence concentrates at a single nonsynonymous substitution (L139I) that is restricted to high elevation and enriched in infected high-elevation individuals; *ATP6* diversity is lowest in that same group, consistent with a sweep-like background [6–12, 27, 28]. In parallel, high-elevation adults showed higher ATP content and oligomycin-sensitive Complex V activity, whereas citrate synthase activity did not differ, consistent with an elevation-associated difference in ATP synthase–related energetic readouts. Because this two-population contrast cannot orthogonally separate elevation, *Wolbachia* burden, mitochondrial background, and *ATP6* genotype, these energetic readouts provide observational functional context for the broader cytoplasmic-axis pattern.

From a microbiome perspective, *Wolbachia* complicates interpretation because one intracellular taxon can dominate 16S rRNA gene profiles and inflate apparent between-sample dissimilarities [14, 15, 19–22]. In our data, community separation was strongest when *Wolbachia* was retained and weakened—yet did not disappear—after removing *Wolbachia* ASVs and renormalizing. This pattern indicates compositional dominance by *Wolbachia* together with residual abundance shifts among non-*Wolbachia* taxa, rather than wholesale lineage replacement. Exploratory functional predictions from *Wolbachia*-depleted profiles were therefore treated as hypothesis-generating context rather than direct functional evidence.

Overall, our results support a symbiont-aware framework in which *Wolbachia* variation, mtDNA turnover, and microbiome restructuring are statistically aligned within the same host. This perspective helps explain elevational mtDNA clines without assuming mitochondrial adaptation as the sole driver. Establishing mechanism will require experiments that decouple infection, host background, and environmental drivers, and that quantify transmission and fitness effects across strains and conditions.

## Supplementary Material

Supplementary_material_wrag188

## Data Availability

All sequence data generated or re-analyzed in this study are associated with NCBI BioProject PRJNA720281. Raw 16S rRNA gene amplicon reads are deposited in the SRA under accession numbers SRR35295783–SRR35295862 (*n* = 80). Individual gene sequences are available in GenBank under the following accession ranges: mitochondrial *COI* (PX271309–PX272098), mitochondrial *COII* (PX280628–PX281417), mitochondrial *ATP6* (PX260722–PX260801), mitochondrial *ATP8* (PX260802–PX260881), nuclear 28S rRNA gene (PX248778–PX249552 and PZ251272–PZ251286), and *Wolbachia* MLST sequences (PX276818–PX276842). Pooled shotgun metagenomic data re-analyzed as an independent background comparison are associated with the same BioProject. All other data supporting the findings of this study are available within the article and its Supplementary Information files, Supplementary Tables, and DataSheets.
