# Supplementary material for: Wolbachia-centered cytoplasmic axis links elevational mitochondrial DNA turnover with microbiome restructuring
Source: ISME J. 2026 Jul 17;20(1):wrag188. doi: 10.1093/ismejo/wrag188 (PMC13453311; doi:10.1093/ismejo/wrag188)
Supplement: Supplementary file 1 [file supplementary_material_wrag188.zip › Supplementary-ISME.docx]

**Additional file 1 (XLSX):** Tables S1–S10 include site metadata and environmental variables, primers and PCR/qPCR conditions, *wsp* subtyping references, sequencing IDs and accession mapping, *Wolbachia* infection rates and molecular typing, mitochondrial/nuclear diversity summaries, 16S rRNA gene community statistics, *ATP6* codon-model tests, and scale-specific integrated model outputs.

**Additional file 2 (PDF):** Figures S1–S12 provide supporting analyses of 16S rRNA gene sequencing depth, within-population *Wolbachia* titer variation, environmental screening and focal model comparisons, *COI*+*COII* haplotype structure, mtDNA diversity rarefaction and sliding-window summaries, *ATP8* variation, NMDS/PERMDISP sensitivity analyses, *Wolbachia*-depleted ASV distributions, co-abundance module differences, module–titer associations, and exploratory functional profiles with pooled metagenomic background comparison.

**DataSheet 1 (XLSX):** Individual-level metadata, *Wolbachia* infection status, qPCR-derived relative burden, strain-typing information, sample-to-accession mapping, and 16S rRNA gene amplicon read-count summaries.

**DataSheet 2 (XLSX):** Filtered 16S rRNA gene amplicon ASV count table with taxonomic assignments. The *Wolbachia*-depleted table was derived from this filtered table by removing *Wolbachia*-assigned ASVs and renormalizing the remaining bacterial abundances.

**DataSheet 3 (XLSX):** Environmental-variable summaries, multicollinearity/VIF diagnostics, and focal elevation-only, UVA-only, and elevation + UVA model outputs.

**Wolbachia-centered cytoplasmic axis links elevational mitochondrial DNA turnover with microbiome restructuring
Running title:** *Wolbachia* cytoplasmic axis

Yong Zhang¹, Xueyu Huang¹, Qiangkun Li¹, Yuhang Ruan¹, Yanhua Long¹^,^³*, Shouke Zhang²*, Yunqiu Yang¹*

¹State Key Laboratory for Tea Plant Germplasm Innovation and Resource Utilization; Key Laboratory of Tea Biology and Tea Processing, Ministry of Agriculture and Rural Affairs, Anhui Agricultural University, Hefei 230036, Anhui, China
²State Key Laboratory for Development and Utilization of Forest Food Resources, Zhejiang A&F University, Hangzhou 311300, Zhejiang, China
³College of Life Science, Anhui Agricultural University, Hefei 230036, Anhui, China

*Corresponding authors:

Yunqiu Yang, State Key Laboratory for Tea Plant Germplasm Innovation and Resource Utilization; Key Laboratory of Tea Biology and Tea Processing, Ministry of Agriculture and Rural Affairs, Anhui Agricultural University, No. 130 West Changjiang Road, Shushan District, Hefei 230036, Anhui, China. Email: yangyunqiu@ahau.edu.cn

Shouke Zhang, State Key Laboratory for Development and Utilization of Forest Food Resources, Zhejiang A&F University, No. 666 Wusu Street, Lin'an District, Hangzhou 311300, Zhejiang, China. Email: socrazhang@zafu.edu.cn

Yanhua Long, College of Life Science, Anhui Agricultural University, No. 130 West Changjiang Road, Shushan District, Hefei 230036, Anhui, China. Email: longyanhua@ahau.edu.cn.

**Supplementary information**

**Additional file 1**: Supplementary Tables (XLSX)
**Table S1.** Overview of sampling locations, elevation zones, and site-level environmental variables for *Wolbachia* prevalence analyses in tea green leafhopper (TGLH) populations across mainland China

**Table S2.** Primers and PCR/qPCR conditions used in this study

**Table S3.** Reference *wsp* gene profiles and *Wolbachia* subtype classification

**Table S4.** Sample metadata, sequencing IDs, and 16S rRNA gene sequencing of TGLH across elevation and *Wolbachia* infection groups

**Table S5.** *Wolbachia* infection rates and molecular typing in wild TGLH populations

**Table S6.** Summary of mitochondrial and nuclear genetic diversity indices, neutrality tests, and mtDNA patterns across the full gradient, low–mid–high elevation zones, and low/high endpoint groups in TGLH populations

**Table S7.** Codon-model comparisons for *ATP6*, with WIHigh specified as the foreground group where applicable

**Table S8.** Pairwise PERMANOVA summaries for Bray–Curtis 16S rRNA gene community profiles in the *Wolbachia*-retained and *Wolbachia*-depleted ASV tables.

**Table S9**. SEM summary: statistical alignment among elevation/UVA, *Wolbachia* load, mtDNA diversity, and microbiome structure

**Table S10.** Scale-specific integrated model outputs linking mountain-gradient variables, *Wolbachia* load/abundance, mtDNA diversity, and microbiome structure in both *Wolbachia*-retained and *Wolbachia*-depleted profiles

**Additional file 2**: Supplementary Figures (PDF).

**Figure S1. Rarefaction curves for 16S rRNA gene amplicon sequencing.**

(**A**) Rarefaction curves based on observed ASV from the filtered *Wolbachia*-retained ASV table after removal of chloroplast, mitochondrial, non-bacterial, and extremely low-abundance ASVs.

(**B**) Rarefaction curves based on the *Wolbachia*-depleted ASV table after removal of *Wolbachia*-assigned ASVs. Curves are shown for the 80 individuals used in the microbiome analyses and were used to visualize sequencing-depth coverage for the downstream retained and depleted community tables.

**Figure S2. Within-population variation in qPCR-derived relative *Wolbachia* titer among infected populations.**

Individual-level log₂-transformed qPCR-derived relative *Wolbachia* titer values are shown for infected populations with qPCR-based titer estimates. Populations are ordered by elevation and grouped into high (≥1,000 m), mid (400–1,000 m), and low (<400 m) elevation zones. Black points represent individual *wsp*-positive insects, and colored vertical bars indicate the observed within-population range.

**Figure S3. Environmental screening and focal model comparison for *Wolbachia* infection and site-level mean relative titer.**

(**A**) Single-predictor model performance for *Wolbachia* infection prevalence across the evaluated mountain-climate variables. Infection was modeled using site-level binomial GLMs with infected and uninfected counts as the response, and model performance was summarized using Tjur’s *R*².

(**B**) Single-predictor model performance for site-level mean qPCR-derived relative *Wolbachia* titer. Site-level mean relative titer was calculated from *Wolbachia*-infected individuals within each population and analyzed using weighted linear models, with model performance summarized using *R*².

(**C**) Focal model comparison for *Wolbachia* infection prevalence among elevation-only, UVA-only, and elevation + UVA models.

(**D**) Focal model comparison for site-level mean relative *Wolbachia* titer among elevation-only, UVA-only, and elevation + UVA models.

UVA, ultraviolet A radiation; UVB, ultraviolet B radiation; ETR, extreme temperature range; MTR, mean temperature range; P, surface pressure; T, mean temperature; TAP, total annual precipitation; RH, relative humidity. Detailed coefficients, P values, AIC values, ΔAIC values, sample sizes, and VIF outputs are provided in DataSheet 3.

**Figure S4. Full *COI*+*COII* haplotype network and haplotype dominance summaries.**

(**A**) Full TCS haplotype network based on all concatenated *COI*+*COII* haplotypes in the low/high endpoint dataset. Node size is proportional to haplotype frequency, and colors within nodes indicate contributions from the four elevation–infection groups: WIHigh, WILow, WUHigh, and WULow.

(**B**) Haplotype dominance summaries across the four elevation–infection groups, including Hap1 frequency, Shannon Index, and Berger–Parker dominance index calculated from *COI*+*COII* haplotype frequencies within each group.

**Figure S5. Rarefaction-based mtDNA diversity comparisons across elevation–infection groups.**

(**A**) Haplotype diversity (Hd) and (**B**) nucleotide diversity (π) were recalculated for the concatenated *COI*+*COII* dataset after rarefying each elevation–infection group to 60 individuals over 600 iterations. Values show mean ± SD across resamples.

**Figure S6. Sliding-window nucleotide diversity across the concatenated *COI*+*COII* region.**

Sliding-window nucleotide diversity (π) was calculated across the concatenated *COI*+*COII* alignment for WIHigh, WILow, WUHigh, and WULow using a 100-bp window and 25-bp step. WIHigh showed reduced nucleotide diversity across much of the concatenated region.

**Figure S7. *ATP8* divergence between high- and low-elevation samples.**

Per-site *F*_ST_ values across *ATP8* were calculated between high- and low-elevation samples at the nucleotide and amino-acid levels. DNA-level divergence was observed across *ATP8*, whereas amino-acid-level divergence was limited. The only amino-acid substitution, I17T, was detected in 2 of 80 sequences and showed no significant enrichment.

**Figure S8. NMDS sensitivity analysis of Bray–Curtis 16S rRNA gene profiles.**

NMDS ordinations based on Bray–Curtis dissimilarities were generated for the *Wolbachia*-retained (**A**) and *Wolbachia*-depleted (**B**) ASV tables. Points represent individual samples, colors indicate the four elevation–infection groups, and ellipses summarize group dispersion. Stress values are shown in each panel. PERMDISP did not detect significant differences in multivariate dispersion for either the *Wolbachia*-retained table (F = 0.1, df₁ = 3, df₂ = 76, P = 0.966) or the *Wolbachia*-depleted table (F = 2.7, df₁ = 3, df₂ = 76, P = 0.155).

**Figure S9. Phylogeny-resolved distribution of *Wolbachia*-depleted ASVs.**

ASVs retained after removing *Wolbachia*-assigned ASVs are shown in a phylogeny-resolved layout. Tip colors indicate bacterial phyla, and point size indicates mean relative abundance across all samples. Outer abundance tracks show the scaled relative abundance of each ASV across WIHigh, WILow, WUHigh, and WULow.

**Figure S10. Differential abundance of major co-abundance modules between WIHigh and WILow.**

Relative abundance of major co-abundance modules inferred from the *Wolbachia*-depleted genus-level network was compared between infected high-elevation and infected low-elevation individuals. Module abundance was calculated as the summed relative abundance of member genera within each module. Panels show Module 62 (**A**), Module 36 (**B**), Module 69 (**C**), Module 33 (**D**), Module 30 (**E**), Module 21 (**F**), Module 8 (**G**), and Module 13 (**H**). Differences between WIHigh and WILow were tested using Welch’s t-tests with Benjamini–Hochberg correction. Module 8 was higher in WIHigh, whereas Module 33 was higher in WILow. WIHigh, *Wolbachia*-infected high-elevation individuals; WILow, *Wolbachia*-infected low-elevation individuals. ns, not significant; * adjusted *P* < 0.05; ** adjusted *P* < 0.01.

**Figure S11. Exploratory associations between co-abundance module abundance and qPCR-derived *Wolbachia* relative titer.**

Exploratory module–titer associations were assessed among infected individuals from WIHigh and WILow. Each panel shows the relationship between qPCR-derived relative *Wolbachia* titer and the summed relative abundance of one major co-abundance module from the *Wolbachia*-depleted genus-level network: Module 62 (**A**), Module 36 (**B**), Module 69 (**C**), Module 33 (**D**), Module 30 (**E**), Module 21 (**F**), Module 8 (**G**), and Module 13 (**H**). Points represent individual infected insects, colors indicate WIHigh and WILow, and fitted lines with confidence bands are shown for visualization. Pearson’s r and P values are shown in each panel. These analyses were exploratory and were used to evaluate whether non-*Wolbachia* module abundance covaried with qPCR-derived *Wolbachia* relative titer.

**Figure S12. Exploratory functional profiles of *Wolbachia*-depleted bacterial communities and pooled metagenomic background comparison.**

(**A**) Alluvial plot linking predicted KEGG level-3 amino-acid biosynthesis pathways, KEGG Orthologues, contributing bacterial genera, and elevation–infection groups based on the *Wolbachia*-depleted 16S rRNA gene profiles. The analysis was used to provide exploratory, hypothesis-generating functional context rather than direct functional validation.

(**B**) Pooled shotgun metagenomic background comparison for the isoleucine biosynthesis module M00570. Heatmaps show the distribution of selected KEGG Orthologues across low- and high-elevation pooled metagenomes and representative bacterial contributors.

(**C**) Pooled shotgun metagenomic background comparison for the leucine biosynthesis module M00432. Heatmaps show the distribution of selected KEGG Orthologues across low- and high-elevation pooled metagenomes and representative bacterial contributors. L1–L3 and H1–H3 indicate low- and high-elevation pooled metagenomic libraries, respectively. Bacterial abbreviations are shown in the panel. These pooled metagenomes were used as an independent background comparison and not as individual-level validation of the 80-sample 16S rRNA gene-based functional predictions.
